# Experimental strategies for imaging bioparticles with femtosecond hard X-ray pulses

**DOI:** 10.1107/S2052252517003591

**Published:** 2017-04-07

**Authors:** Benedikt J. Daurer, Kenta Okamoto, Johan Bielecki, Filipe R. N. C. Maia, Kerstin Mühlig, M. Marvin Seibert, Max F. Hantke, Carl Nettelblad, W. Henry Benner, Martin Svenda, Nicuşor Tîmneanu, Tomas Ekeberg, N. Duane Loh, Alberto Pietrini, Alessandro Zani, Asawari D. Rath, Daniel Westphal, Richard A. Kirian, Salah Awel, Max O. Wiedorn, Gijs van der Schot, Gunilla H. Carlsson, Dirk Hasse, Jonas A. Sellberg, Anton Barty, Jakob Andreasson, Sébastien Boutet, Garth Williams, Jason Koglin, Inger Andersson, Janos Hajdu, Daniel S. D. Larsson

**Affiliations:** a Laboratory of Molecular Biophysics, Department of Cell and Molecular Biology, Uppsala University, Husargatan 3 (Box 596), SE-751 24 Uppsala, Sweden; b NERSC, Lawrence Berkeley National Laboratory, Berkeley, California, USA; c Division of Scientific Computing, Department of Information Technology, Science for Life Laboratory, Uppsala University, Lägerhyddsvägen 2 (Box 337), SE-751 05 Uppsala, Sweden; d Physical and Life Sciences Directorate, Lawrence Livermore National Laboratory, 7000 East Avenue, Livermore, CA 94550, USA; e Molecular and Condensed Matter Physics, Department of Physics and Astronomy, Uppsala University, Lägerhyddsvägen 1 (Box 516), SE-751 20 Uppsala, Sweden; f Center for Free-Electron Laser Science, DESY, Notkestrasse 85, 22607 Hamburg, Germany; g Centre for BioImaging Sciences, National University of Singapore, Singapore; h Bhabha Atomic Research Center, Mumbai 400 085, India; i Department of Physics, Arizona State University, Tempe, AZ 85287, USA; j The Hamburg Center for Ultrafast Imaging, University of Hamburg, Luruper Chaussee 149, 22761 Hamburg, Germany; k Biomedical and X-ray Physics, Department of Applied Physics, AlbaNova University Center, KTH Royal Institute of Technology, SE-106 91 Stockholm, Sweden; l ELI Beamlines, Institute of Physics, Czech Academy of Science, Na Slovance 2, 182 21 Prague, Czech Republic; m Linac Coherent Light Source, SLAC National Accelerator Laboratory, 2575 Sand Hill Road, Menlo Park, CA 94025, USA; n Brookhaven National Laboratory, 743 Brookhaven Avenue, Upton, NY 11973, USA; o Institute of Physics AS CR, v.v.i., Na Slovance 2, 182 21 Prague 8, Czech Republic

**Keywords:** X-ray diffraction, free-electron laser, flash X-ray imaging, diffraction before destruction, virus, *Omono River virus*, OmRV

## Abstract

This study explores the capabilities of the Coherent X-ray Imaging Instrument at the Linac Coherent Light Source to image small biological samples. The weak signal from small samples puts a significant demand on the experiment. Aerosolized *Omono River virus* particles of ∼40 nm in diameter were injected into the submicrometre X-ray focus at a reduced pressure. Diffraction patterns were recorded on two area detectors. The statistical nature of the measurements from many individual particles provided information about the intensity profile of the X-ray beam, phase variations in the wavefront and the size distribution of the injected particles. The results point to a wider than expected size distribution (from ∼35 to ∼300 nm in diameter). This is likely to be owing to nonvolatile contaminants from larger droplets during aerosolization and droplet evaporation. The results suggest that the concentration of nonvolatile contaminants and the ratio between the volumes of the initial droplet and the sample particles is critical in such studies. The maximum beam intensity in the focus was found to be 1.9 × 10^12^ photons per µm^2^ per pulse. The full-width of the focus at half-maximum was estimated to be 500 nm (assuming 20% beamline transmission), and this width is larger than expected. Under these conditions, the diffraction signal from a sample-sized particle remained above the average background to a resolution of 4.25 nm. The results suggest that reducing the size of the initial droplets during aerosolization is necessary to bring small particles into the scope of detailed structural studies with X-ray lasers.

## Abstract 2

Facilitating the very short and intense pulses from an X-ray laser for the purpose of imaging small bioparticles carries the potential for structure determination at atomic resolution without the need for crystallization. In this study, experimental strategies for this idea are explored based on data collected at the Linac Coherent Light Source from 40 nm virus particles injected into a hard X-ray beam.

## 1. Introduction   

Imaging of biological macromolecules using conventional methods is ultimately limited by radiation damage owing to the energy deposited in the sample by the probing beam (Henderson, 1995 ▸). Flash X-ray imaging (FXI) utilizes femtosecond X-ray pulses produced by X-ray free-electron lasers (XFELs) to image single particles based on the principle of ‘diffraction before destruction’, in which the very short probing pulse outruns key processes in radiation damage (Neutze *et al.*, 2000 ▸). Single-particle methods such as FXI have the potential to resolve yet unknown structural aspects in molecular biology, such as the asymmetric internal features of icosahedral viruses (Song *et al.*, 2008 ▸; Ekeberg *et al.*, 2015 ▸).

FXI was first demonstrated using solid targets (Chapman *et al.*, 2006 ▸) at the Free-electron Laser Hamburg (FLASH; Ayvazyan *et al.*, 2006 ▸) and has since been applied to a variety of biological samples at the Linac Coherent Light Source (LCLS; Emma *et al.*, 2010 ▸) and at the SPring-8 Angstrom Compact free electron LAser (SACLA; Ishikawa *et al.*, 2012 ▸). As the method has matured (Aquila *et al.*, 2015 ▸; Miao *et al.*, 2015 ▸), there has been a steady improvement in the achievable resolution and ever smaller samples have been studied, ranging from small cells (∼1 µm; Seibert *et al.*, 2010 ▸; Kimura *et al.*, 2014 ▸; van der Schot *et al.*, 2015 ▸; Fan *et al.*, 2016 ▸) to giant viruses (∼450 nm; Seibert *et al.*, 2011 ▸; Ekeberg *et al.*, 2015 ▸), cell organelles (∼115 nm; Hantke *et al.*, 2014 ▸) and intermediate-sized viruses (∼70 nm; Munke *et al.*, 2016 ▸).

Most recent FXI experiments were performed on the soft X-ray beamline for atomic, molecular and optical experiments (AMO; Bozek, 2009 ▸) at the LCLS using photon energies of between 0.5 and 2 keV. Achieving atomic resolution with FXI requires the use of harder X-rays. The Coherent X-ray Imaging (CXI) instrument at the LCLS (Boutet & Williams, 2010 ▸; Liang *et al.*, 2015 ▸) can operate in the range 5–11 keV. Increasing the photon energy improves the theoretical resolution limit but comes with challenges. Operating at hard X-ray energies results in fewer incident photons in the pulse since LCLS gives a rather constant integrated pulse energy and fewer elastically scattered photons owing to a lower scattering cross-section. This puts elevated demands on the beamline optics. The X-rays have to be shaped into a tightly focused beam to maximize the photon fluence through the sample, and extraneous background photons need to be minimized to avoid overwhelming the sparse diffraction signal from the sample. Furthermore, a tightly focused beam results in a smaller illuminated volume and therefore results in lower hit ratios, which makes accurate sample delivery important. Different strategies for sample delivery have been tested. Aerosol injection (Bogan *et al.*, 2008 ▸; Seibert *et al.*, 2011 ▸; Hantke *et al.*, 2014 ▸; van der Schot *et al.*, 2015 ▸; Ekeberg *et al.*, 2015 ▸; Munke *et al.*, 2016 ▸) has shown a lot of promise and is a method that reduces potential background to the carrier focusing gas. Alternative delivery methods are depositing samples on silicon nitride membranes (Seibert *et al.*, 2010 ▸; Fan *et al.*, 2016 ▸) or keeping them in a thin water cell (Kimura *et al.*, 2014 ▸).

In this experiment, we explored the capabilities of the CXI instrument for imaging small biological samples in the sub­micrometre focus using a well characterized sample, the ∼40 nm *Omono River virus* (Okamoto *et al.*, 2016 ▸). From a collection of 4555 diffraction patterns, we individually estimated the corresponding particle sizes and found that most injected particles were of spherical shape and followed a wide size distribution. We compared these observations with independent measurements of the particle size in solution and concluded that smaller initial droplets would decrease the polydispersity of the injected particles. In addition, we estimated the incident photon intensity for each diffraction event and derived average properties of the full-power X-ray beam in the focus. Finally, we picked a strong sample-sized diffraction pattern. We compared its diffraction signal with the average background signal as a function of diffraction angle and reconstructed a two-dimensional projection image using standard phase-retrieval techniques.

## 2. Methods   

**Figure 1:**
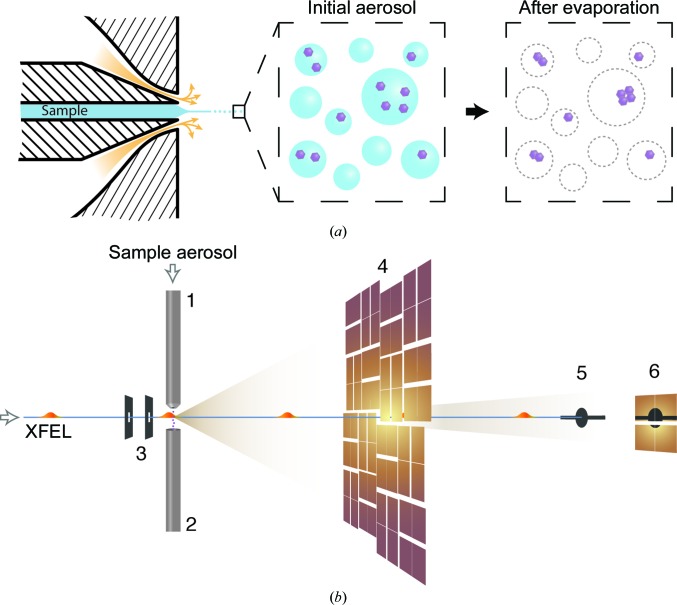
Schematic experimental setup. (*a*) The sample was aerosolized by a gas dynamic virtual nozzle (GDVN). Each droplet could capture zero, one or multiple virus particles. After evaporation, droplets with multiple particles are likely to form aggregates. (*b*) The aerosol was focused by an aerodynamic lens stack (1) into the interaction region. A catcher (2) connected to a turbo vacuum pump removed gas and sample particles beyond the interaction region. Two silicon apertures (3) inside the experimental chamber reduced extraneous background scattering from the beamline. Diffraction patterns were captured with a 2.3 megapixel front detector (4) and a smaller 140 kilopixel back detector (6) positioned approximately 497 and 2400 mm downstream of the interaction region, respectively. A beam stop (5) prevented the direct beam from hitting the back detector.

The measurements were performed in the submicrometre-focus chamber of the CXI instrument (Boutet & Williams, 2010 ▸; Liang *et al.*, 2015 ▸), in which a pair of Kirkpatrick–Baez (KB) mirrors (Siewert *et al.*, 2012 ▸) gives a nominal X-ray focus of 100 nm. The experimental setup is shown in Fig. 1 ▸. The photon energy was 5.5 keV, with a pulse length of 50 fs at 120 Hz. The average pulse energy was 3.29 mJ, with a standard deviation of 0.29 mJ, as measured by a gas monitor upstream of the KB mirrors. A 100 µm thick diamond window with an estimated transmission of 61% at 5.5 keV was situated at the entrance to the interaction chamber (Boutet & Williams, 2010 ▸).

### 2.1. Sample preparation   

*Omono River virus* (OmRV; isolate AK4; Isawa *et al.*, 2011 ▸) was isolated from C6/36 *Aedes albopictus* mosquito cells. Purification was performed at 4°C. The cells were pelleted by centrifugation at 10 000*g *for 15 min and discarded. The remaining culture fluid was concentrated using a centrifugal filtration tube (Vivaspin 20, Sartorius Stedim) at 6000*g*. The sample was layered onto a bed of 30% sucrose in TNE buffer (20 m*M* Tris–HCl, 150 m*M* NaCl, 1 m*M* EDTA pH 7.5) and was pelleted by ultracentrifugation at 28 000 rev min^−1^ (∼140 000*g*) for 3 h (SW 40 Ti, Beckman Coulter). The pellet was resuspended in 1 ml TNE buffer, applied onto a 12 ml preformed continuous 5–50% sucrose gradient in TNE buffer and ultracentrifugated at 18 000 rev min^−1^ (∼58 000*g*) for 18 h (SW 40 Ti, Beckman Coulter). The virus-containing fraction was identified by measuring the absorbance at 280 nm and by SDS–PAGE and was subsequently incubated with an equal volume of detergent (B-PER II, Thermo Scientific) for 30 min with orbital rotation at room temperature. The sample was filtered through a 0.10 µm membrane (Acrodisc 32 mm, Pall Corporation) and ultracentrifuged at 28 000 rev min^−1^ (∼140 000*g*) for 3 h (SW 40 Ti, Beckman Coulter). The pellet was resuspended in 1 ml injection buffer (100 m*M* ammonium acetate pH 7.5) and then repeatedly dialyzed against the same injection buffer.

### 2.2. Sample characterization   

The particle size distribution was validated in the gas phase by scanning mobility particle sizer (SMPS) spectrometry (Electrospray Aerosol Generator model 3480, Scanning Mobility Particle Sizer model 3081 and Condensation Particle Counter model 3787; all from TSI Inc.) and by electron-microscopy (EM) imaging of negative-stained (2.0% uranyl acetate) samples (Quanta SEM in STEM mode, FEI Company).

### 2.3. Sample injection   

The sample was kept in 100 m*M* ammonium acetate at a particle density of 1.0 × 10^13^ particles ml^−1^. Prior to the experiment it was filtered and diluted five times. The sample was aerosolized using a gas dynamic virtual nozzle (GDVN; DePonte *et al.*, 2008 ▸; Gañán-Calvo *et al.*, 2010 ▸) in electroflow focusing mode (Gañán-Calvo *et al.*, 2006 ▸). A high-voltage power supply was connected to the liquid in the line between the sample reservoir and the GDVN. The sample reservoir, liquid lines and nozzle holder were electrically insulated and the aerosol relaxation chamber was at zero potential and acted as the counter electrode. A voltage of −1.5 kV was applied to the solution and currents of 27–35 µA flowed through the liquid and *via* charged aerosol droplets. The sample flow rate was set to 0.3–1 µl min^−1^ by pressurizing the reservoir with helium (517–1310 kPa). Helium was also used as the sheath gas in the GDVN (2340–2790 kPa). The aerosol was focused by an aerodynamic lens stack driven by differential pumping into an ∼20 µm wide particle beam (as described in Hantke *et al.*, 2014 ▸). The pressure before the lens was 200 Pa and the pressure in the sample chamber was 0.01 Pa. A coarse injector alignment was performed using optical feedback. The alignment was subsequently optimized by scanning the injector in small steps perpendicular and transversal to the XFEL beam while monitoring the X-ray diffraction signal.

### 2.4. Additional instrumentation   

In addition to the common beamline components (Boutet & Williams, 2010 ▸), two square silicon apertures with apodized edges (Silson Ltd) were installed inside the sample chamber in order to reduce extraneous scattering from the X-ray optics and slits. One aperture, with an 800 µm opening, was placed ∼50 mm upstream of the interaction point. The other, with a 500 µm opening, was placed just upstream (5–10 mm) of the interaction point. Their positions were tuned to minimize beamline scattering.

An array of ion time-of-flight (iToF) detectors was installed inside the chamber for online hit-finding by monitoring the ion fragments from particles interacting with the X-ray beam.

### 2.5. Data collection   

The main detector of the CXI instrument is a 2.3 megapixel camera based on the Cornell–SLAC pixel-array detector (CSPAD; Hart *et al.*, 2012 ▸) design. For this experiment, a version 1.5 camera with upgraded electronics, application-specific integrated circuits (ASICs) and firmware (Carini *et al.*, 2014 ▸) was positioned 497 mm downstream of the interaction region and a smaller 140 kilopixel camera was installed 2.4 m downstream of the interaction region to cover the lower diffraction angles. Both detectors had pixels with an edge length of 110 µm and a quantum efficiency of 95.5% at 5.5 keV owing to a 1.2 µm aluminium coating (Hart *et al.*, 2012 ▸). The direct beam passed through a 100 pixel square hole at the centre of the front detector and ended up on a beam stop placed in front of the back detector, leaving a shadow on the diffraction patterns.

The data from both CSPAD detectors together with other metadata were read at 120 Hz and stored by the LCLS data-acquisition system as XTC files. A reference data set was collected in between sample runs on buffer to determine the injection background level. We flushed sample lines for 5 min to minimize the risk of cross-contamination.

### 2.6. Real-time monitoring   

We developed a real-time analysis tool for data selection, injection diagnostics and visualization of diffraction patterns (Daurer *et al.*, 2016 ▸). The software had direct access to the LCLS experimental data stream through the interactive Python interface of the *psana* framework (Damiani *et al.*, 2016 ▸).

### 2.7. Data reduction and corrections   

Conversion of the raw data into the CXI file format (Maia, 2012 ▸) was performed using the *Cheetah* software package (Barty *et al.*, 2014 ▸). For the detectors, the conversion involved a calibration based on averaging dark frames, a per-ASIC common-mode correction using the median of unbonded and shadowed pixels as a reference, and masking saturated, hot and bad pixels. An additional mask was established that blocked strong background scattering.

The patterns were reduced to the subset containing particle hits only. These hits were identified by counting the number of illuminated (lit) pixels (*i.e.* receiving at least one photon) on the back detector in each frame. Counts exceeding a hit score of at least 600 lit pixels were regarded as hits.

For the selected particle hits, the raw pixel data were assembled (*i.e.* resampled onto a grid with physical coordinates) based on given metrology and additional adjustments using diffraction patterns with very strong scattering. Finally, data from both detectors were combined by interpolating the back detector on a grid defined by the pixel spacing of the front detector.

### 2.8. Classification of particles based on diffraction data   

To classify hits, the intensities at low diffraction angles covered by the back detector were fitted to the calculated scattering from a homogeneous sphere. Similar model-based approaches for sizing have already been described in Loh *et al.* (2013 ▸) and Hantke *et al.* (2014 ▸). Considering the geometry of a typical scattering experiment and given an incident photon intensity *I* ^0^, the scattered intensity from a sphere of diameter *d* measured in a given detector pixel *i* with reciprocal scattering vector **q**_*i*_ can be described as 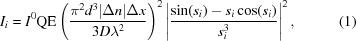
where 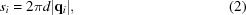
λ is the photon wavelength, *D* is the detector distance, QE is the detector quantum efficiency, Δ*x* is the detector pixel size and (1 − Δ*n*) is the refractive index of the particle. The refractive index used was based on a typical atomic composition of virus particles as reported for poliovirus by Molla *et al.* (1991 ▸) and assuming a mass density of 1340 kg m^−3^ (Dans *et al.*, 1966 ▸).

We used a four-step algorithm for model-based classification of diffraction data.

(i) Find the centre of diffraction (*x*, *y*) by maximizing the pairwise cross-correlation of nonmasked centrosymmetric pixels (assuming Friedel symmetry).
(ii) Find a rough estimate of the particle size *d* by maximizing the Pearson correlation between the modelled intensities *I_i_*(*d*) and the measured intensities *n_i_*, since the correlation is insensitive to *I* ^0^.
(iii) Find a rough estimate of the incident photon intensity *I* ^0^ by minimizing the sum of squared differences between *I_i_*(*I* ^0^) and *n_i_*.
(iv) Refine all estimates *x*, *y*, *d* and *I* ^0^ by minimizing the sum of squared differences between *I_i_*(*x*, *y*, *d*, *I* ^0^) and *n_i_*.

This procedure was used on the back-detector data to classify hits based on particle size and incident photon intensity using the implementation of the Levenberg–Marquardt algorithm in SciPy for the least-squares optimization. For the comparison of modelled and measured diffraction intensities, a radially symmetric mask was used to avoid contributions from high-angle intensities where the measured diffraction pattern is represented less accurately by the diffraction of a sphere. To reduce the number of misclassifications, each assessment was manually checked by visual comparison of the model and the data. The entire classification procedure has been adapted for real-time analysis and is available within the *Hummingbird* toolbox (Daurer *et al.*, 2016 ▸).

### 2.9. Validation of the sphere-fitting approach   

To test the validity of the sphere-fitting approach to particle classification, we simulated a diffraction data set of spheres with sizes between 1 and 300 nm and incident photon intensities ranging from 5 × 10^8^ to 5 × 10^12^  photons µm^−2^. The diffraction patterns were calculated with help of the *Condor* software package (Hantke *et al.*, 2016 ▸) with parameters matching those of the experiment described in this paper (including the mask). Each pattern was sampled by Poisson statistics, a random experimentally measured background frame was added and the centre position was shifted randomly. The simulated data with ten different samples of each pair of particle size and intensity was processed using the same procedure as described in §[Sec sec2.8]2.8, namely calculating a hit score, finding centre positions and estimating particle sizes and incident photon intensities based on fitting to the sphere model.

**Figure 2:**
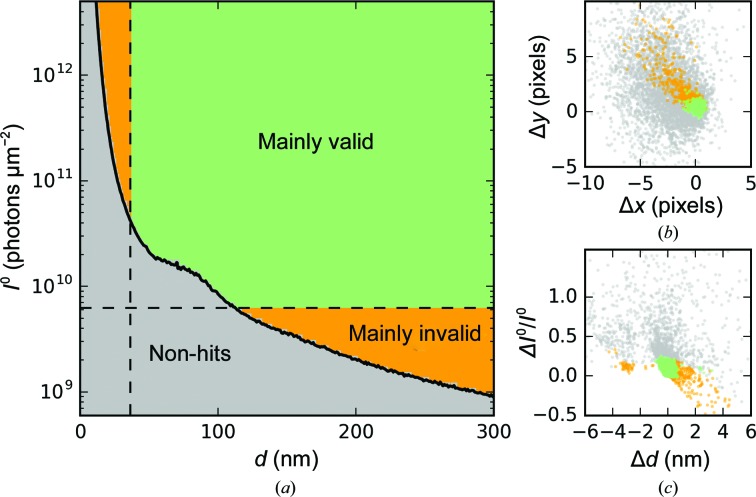
Validation of the particle-classification procedure based on simulation of spheres with different particle sizes and photon intensities. (*a*) Classification of patterns as nonhits (grey area) and hits (above the black solid line) as function of particle size and intensity. Data points with strong deviations in the size and intensity estimates are depicted in orange; the rest are shown in green and separated by black dashed lines. (*b*) Distribution of errors in the diffraction centre. (*c*) Distribution of errors in particle size and intensity (normalized to the simulated intensity). The colour-coding in (*b*) and (*c*) follows the categories shown in (*a*). A statistical summary of the green distributions is given in Table 1 ▸.

The estimated values for the diffraction centre (*x*_fit_, *y*_fit_), the particle size *d*_fit_ and the intensity *I*_fit_^0^ were averaged across the ten different samples and compared with the ground truth of the simulation. Fig. 2 ▸(*a*) shows regions with small errors in green (and summarized in Table 1 ▸). For particles with a diameter below 35 nm and for intensities below 5.86 × 10^9^  photons µm^−2^ the estimates deviated significantly from the ground truth and are shown in orange. Combinations that resulted in diffraction below the hit-finding limit of 600 lit pixels (solid black line) are shown in grey. The distribution of errors is also shown in Figs. 2 ▸(*b*) and 2 ▸(*c*), coloured according to the categories in Fig. 2 ▸(*a*).

### 2.10. Reconstruction of the beam profile   

Under the assumption that the intensity in an azimuthally averaged X-ray beam focus falls off monotonically as a function of the transverse distance to the optical axis, the average profile can be recovered. This is performed by sorting the estimated observed incident photon intensities *I*_*k*_^0^ = *I*_1_^0^ > *I*_2_^0^ > *I*_3_^0^, …, *I*_*N*_^0^ according to their values and thereby introducing an index *k* running from 1 to *N*, the total number of observations. Assuming that the injected particles uniformly sample this idealized beam, the indices *k* should be proportional to an area π*r_k_* ^2^, where *r_k_* is the radial distance from the optical axis. Replacing *k* with β^2^ *k*, where β is an unknown scaling constant, and given an estimate for the pulse energy in the focus *E*_focus_, the normalization 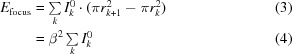
defines the scaling constant 
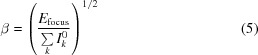
and relates the sorted intensities *I*_*k*_^0^ to distances *r_k_* = β/(π*k*)^1/2^.

### 2.11. Phasing   

To recover the phase of a single two-dimensional diffraction pattern, we used the image-reconstruction package *HAWK* (Maia *et al.*, 2010 ▸) with hybrid input–output (HIO; Fienup, 1978 ▸) and error-reduction (ER; Gerchberg & Saxton, 1972 ▸) algorithms and a static support. For validation purposes, we ran 5000 independent reconstructions (random guess of initial phase) and calculated the phase-retrieval transfer function (PRTF; Chapman *et al.*, 2006 ▸).

### 2.12. Access to data and algorithms   

The data set used in this study has been deposited in the Coherent X-ray Imaging Data Base (CXIDB) and is available at http://cxidb.org/ under ID 56. The data entry contains a collection of hits and background frames together with relevant metadata in CXI file format. In addition, processed data files (*e.g.* size/intensity estimates) and supplementary data (*e.g.* masks, geometry files) are deposited. A collection of software tools together with a comprehensive description of our data analysis has been packaged and made available in a public repository (https://github.com/FXIhub/cxic9714-analysis).

## 3. Results and discussion   

### 3.1. Detector characterization   

**Figure 3:**
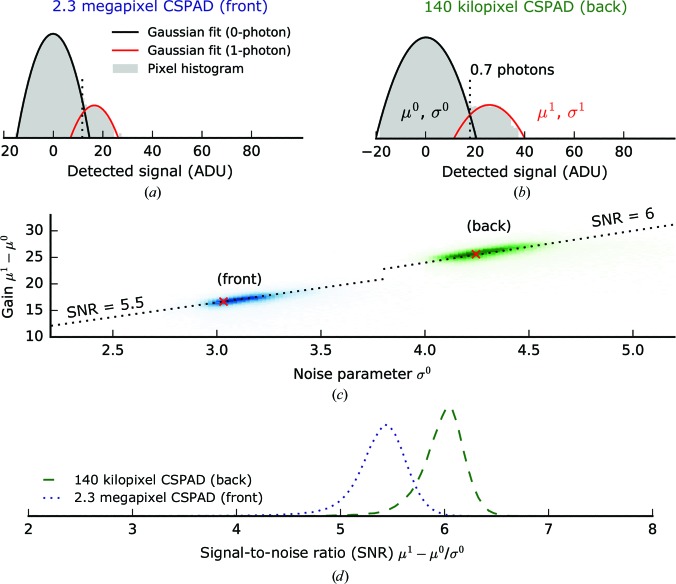
Pixel-wise characterization of the CSPAD detectors. (*a*, *b*) Normalized histograms (log scale) for two representative pixels integrated across all detected frames with Gaussian functions fitted to the zero- and one-photon peaks. The black dotted lines correspond to the 0.7 photon threshold used for counting. (*c*) Two-dimensional histograms of noise and gain estimates placed on grids of 100 × 100 pixels. The red crosses correspond to the values of μ^0^, μ^1^ and σ^0^ shown in the pixel histograms above. (*d*) Signal-to-noise ratio for all pixels shown as normalized histograms (linear scale).

Both CSPAD detectors allowed single-photon counting at 5.5 keV photon energy. For each pixel, a normalized intensity histogram over all of the collected experimental data showed that the one-photon peak was separated from the zero-photon peak (detector noise) (see Figs. 3 ▸ *a* and 3 ▸ *b*). Fitting Gaussians to both the zero-photon and the one-photon peaks of all pixel histograms, gain μ^1^ − μ^0^ and noise σ^0^ parameters were determined for most pixels on both detectors.

The two-dimensional histograms shown in Fig. 3 ▸(*c*) indicate a linear relationship between gain and noise describing a signal-to-noise ratio,
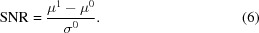
The performance of both CSPAD detectors were characterized individually, giving rise to average SNR values of 6.0 (back) and 5.5 (front). Histograms (normalized to unity) of all SNR values are shown in Fig. 3 ▸(*d*).

The fitting procedure for the gain estimation was successful for 97.4% of the back pixels and 85.1% of the front pixels, which is likely to be related to poor statistics on certain areas of the detector. Therefore, we used the average SNR together with estimates of the noise parameter σ*_i_* ^0^ to construct a complete pixel-wise gain map, 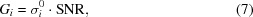
which we used to convert the measured intensities *n_i_*, given in arbitrary analogue-to-digital units (ADU), into photon counts,
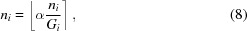
where α equals 0 when *n_i_*/*G_i_* < 0.7 and otherwise equals 1, and 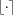 is a rounding operator converting any value to its closest integer. The value of 0.7 was chosen empirically to minimize the rate of false positives and negatives. For the back detector, this corresponds to 4.2σ^0^ and 3.4σ^1^, which gives a false-positive rate of 1 in 37 465 and a false-negative rate of 1 in 1728. For the front detector, this corresponds to 3.85σ^0^ and 2.8σ^1^, which gives a false-positive rate of 1 in 8466 and a false-negative rate of 1 in 202.

### 3.2. Overview of collected data   

**Figure 4:**
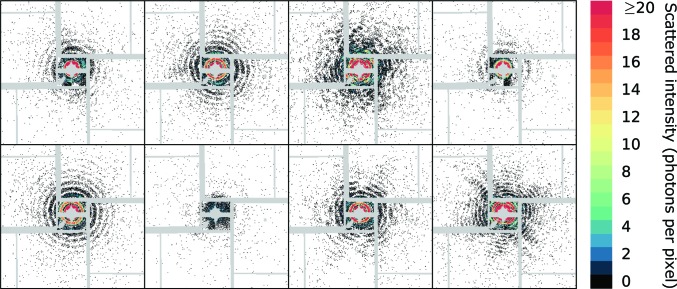
Eight representative hits showing a variety of different diffraction patterns. Masked values are shown in grey.

Within a total collection time of about 235 min, we recorded 1.2 million diffraction patterns while OmRV was injected. From those, 697 028 patterns were collected under similar sample-injection conditions. Using the lit-pixel counter, 5771 patterns were identified as hits, equivalent to an average hit ratio of 0.83%. After classification using the automated scheme described in §[Sec sec2.8]2.8, particle sizes and incident photon intensities were assigned to 4555 patterns. A selection of assembled (back and front) diffraction patterns is shown in Fig. 4 ▸; many diffraction patterns appear to come from spherical particles and variations in both particle size and intensity are noticeable.

**Figure 5:**
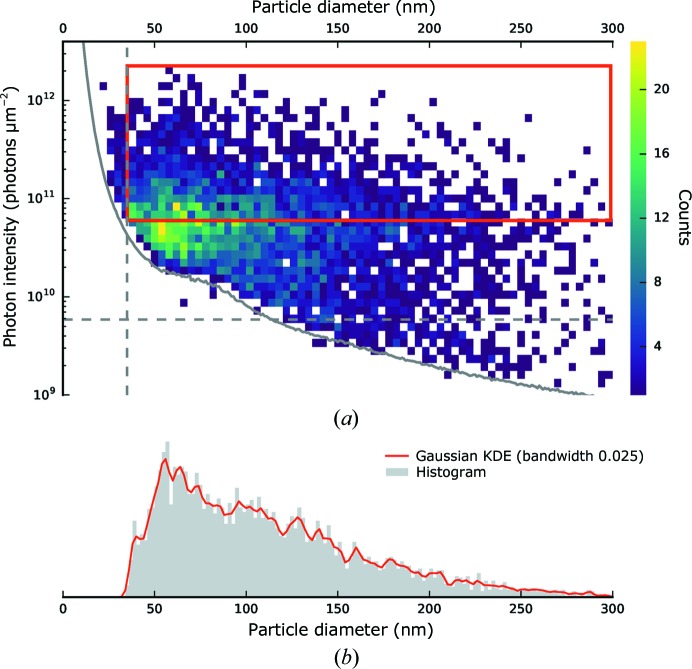
Classification of hits based on fitting to a sphere diffraction model. (*a*) Distribution of particle sizes and incident photon intensities shown as a two-dimensional histogram. The solid/dashed grey lines indicate the same detection/classification limits as described in Fig. 2 ▸. The size of a diameter bin was chosen to be 4 nm, while in the intensity direction 50 bins have been logarithmically distributed between 10^9^ and 10^13^ photons µm^−2^. (*b*) Distribution of particle sizes (integrated inside the red rectangle along the vertical direction) shown as a histogram with a bin size of 2 nm and a Gaussian kernel density estimation (KDE) with a bandwidth of 0.025.

The complete distribution of particle sizes and intensities is presented in Fig. 5 ▸. The estimated incident photon intensity covered three orders of magnitude from ∼10^9^ to 1.9 × 10^12^ photons µm^−2^. The estimated particle sizes appeared in a range from 30 to 300 nm, with a slight enrichment of sample-sized particles (∼40 nm) and a maximum at ∼55 nm. This shows that it was possible to detect sample-sized particles among a wider size distribution.

### 3.3. Sample injection   

**Figure 6:**
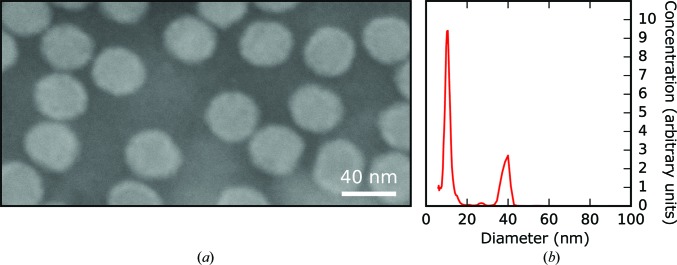
(*a*) Negatively stained EM image of OmRV particles in a buffer of ammonium acetate. (*b*) Size distribution measured using SMPS spectrometry.

As part of the sample characterization prior to the diffraction experiment, the expected particle size of ∼40 nm (Okamoto *et al.*, 2016 ▸) was confirmed by EM imaging of negatively stained particles (Fig. 6 ▸ *a*) and by sizing of aerosolized particles by SMPS spectrometry (Fig. 6 ▸ *b*).

The SMPS measurement used electrospray ionization (ESI), and this produced small droplets of about 150 nm in diameter. The narrow size distribution of these droplets led to a narrow particle peak for the virus at ∼40 nm (Fig. 6 ▸ *b*). This value is in excellent agreement with the EM data. In contrast, GDVNs used at the LCLS produced much larger droplets, and did so over a broader size range (between about 800 and 2000 nm). Larger droplets from GDVNs trap more nonvolatile contaminants than smaller drops from ESI. The significantly broader size distribution of the particles imaged at the LCLS (Fig. 5 ▸ *b*) was attributed to nonvolatile contaminants present in the sample. These contaminants show up clearly as a strong peak at ∼10 nm in the ESI data (Fig. 6 ▸ *b*). The sharpness of this ∼10 nm peak indicates a narrow size distribution for droplets produced by the electrospray process in the SMPS spectrometer. Assuming that these droplets were 150 nm in diameter, the volumetric fraction of impurities in these droplets is estimated to be 0.0003. Extrapolating the volume fraction of contaminants to 800–2000 nm initial droplets, one can expect clusters with diameters of 53–130 nm, similar to the size distribution in Fig. 5 ▸(*b*).

This result emphasizes the need to keep the sample in an ultrapure buffer that only contains volatile species. However, for most biological samples it is difficult to maintain the native state and stability of the particles in such minimal buffers. An alternative is to produce smaller droplets and thereby reduce the amount of trapped contaminants.

### 3.4. Average intensity profile in the X-ray focus   

**Figure 7:**
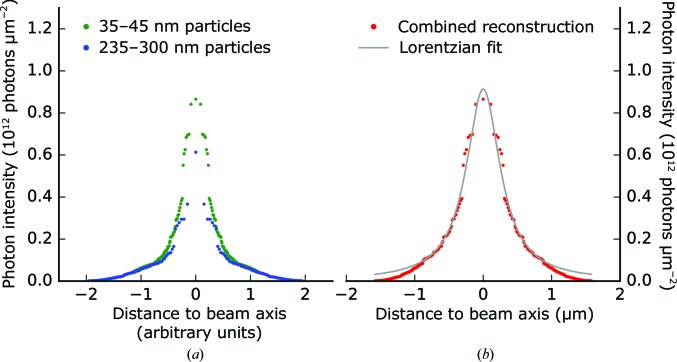
Reconstruction of the average X-ray beam profile in the focus. Injected particles are assumed to sample this profile uniformly. (*a*) Independent reconstructions using intensities that correspond to the smallest detectable particle sizes (35–45 nm) and the largest observed particle sizes (235–300 nm). (*b*) Combined reconstruction using the blue tails and the green centre from (*a*), with the *x* axis being rescaled such that the integrated profile equals a pulse energy in the focus of 0.66 mJ (based on 3.29 mJ measured upstream of the optics and assuming 20% transmission). With this scale, the reconstructed profile has an FWHM of 522 nm. A Lorentzian fit to the profile is shown in grey.

Based on inverting the distribution of intensities shown in Fig. 5 ▸, we reconstructed the average beam profile of the X-ray focus. The inversion assumed a uniform sampling of an ideal cylindrical beam, with the intensity falling off monotonically with distance from the beam axis. Since the probe size limits the resolution of the reconstruction, selected intensities were chosen based on particle sizes in the 35–45 nm range (green profile in Fig. 7 ▸ *a*). However, for this size range lower intensities fall below the detection limit. To improve sampling in the low-intensity tails of the profile, an additional lower resolution reconstruction was made selecting for particles in the 235–300 nm range (blue profile in Fig. 7 ▸ *a*). For the final reconstruction, both profiles were combined (Fig. 7 ▸ *b*). The shape of the reconstructed profile was in good agreement with a Lorentzian distribution, suggesting that a significant amount of the total beam power was falling into low-intensity regions.

The absolute scaling of the reconstruction was performed by comparing the integrated area under the curve with an estimate of the total number of photons in the interaction region. Given the measured pulse energy of 3.29 mJ upstream of the focusing optics and assuming a beamline transmission of 20%, the reconstructed beam was estimated to have a full-width at half-maximum (FWHM) of 522 nm. This estimate has a high degree of uncertainty. The pulse energy was monitored for the unfocused beam, but the transmission through the beamline is not known. Overfilling of the KB focusing mirrors at 5.5 keV, the diamond window and the aluminium shield of the detector all contribute to loss of transmission. Some other estimates of the FWHM based on different beamline transmission assumptions are listed in Table 2 ▸. In addition, the maximum radius of the beam sampled by the 300 nm aggregates are listed. Photons in low-intensity regions beyond this radius did not contribute to the estimate of the FWHM at a given beamline transmission.

The reconstructed beam was an order of magnitude smaller than the width of the particle beam, which justifies the assumption of uniform sampling. Furthermore, the Rayleigh length in the focus is expected to be much larger than the particle beam, which justifies the chosen approach of reconstructing the average beam profile.

### 3.5. Local phase variations in the wavefront   

In addition to the interpretation of the beam intensities, the centre position of each diffraction pattern gave information on local phase variations in the wavefront. We observed pixel shifts of up to 35 pixels in both the vertical and horizontal directions. These shifts correspond to different phase tilts in the wavefront interacting with the sample. This could be interpreted as different angles of the incident planar X-ray beam (pulse-to-pulse pointing instability) or a spatial fine structure with local distortions of the wavefront. The different modes of the focus were sampled by the injected particles in proportion to their temporal or spatial extent.

**Figure 8:**
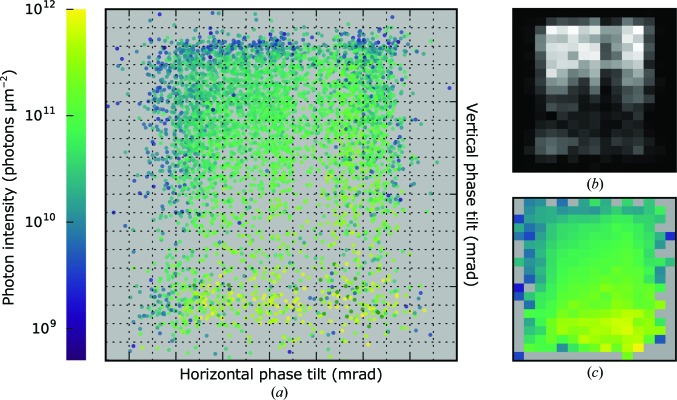
Map of local phase tilts of the wavefront in the focus of the X-ray beam. (*a*) Relative vertical/horizontal deviations from a planar wavefront with estimated photon intensity coded in colour. The horizontal axis spans 1.3 mrad and the vertical axis spans 1.7 mrad. Each box on the grid has dimensions of 0.1 × 0.1 mrad. (*b*) Two-dimensional histogram showing the number of events in each box (linear colour scale with black = 0 and white = 75). (*c*) Map of average photon intensity inside each box coded according to the colour scale on the left.

The distribution of recovered phase tilts is shown in Fig. 8 ▸(*a*) with the corresponding photon intensity values mapped on top. A discretization into boxes of 0.1 × 0.1 mrad shows that some phase tilts appear more frequently than others (top part in the two-dimensional histogram of Fig. 8 ▸ *b*), while other phase tilts correspond to higher/lower values of photon intensity (bottom-right against top-left part in the mean intensity map in Fig. 8 ▸ *c*). Although showing the presence of different phase shifts in the wavefront, it was not possible to map this information onto its spatial location. Nevertheless, this suggests that the average focus of the submicrometre chamber of the CXI instrument has multiple modes, which has also been observed previously in the micrometre focus chamber at the AMO end station (Loh *et al.*, 2013 ▸).

Although the measured intensity distributions were fitted to an azimuthally symmetric Lorentzian in Fig. 7 ▸, the phase-tilt distribution plot in Fig. 8 ▸ suggests that the actual ensemble of pulse profiles may not have this full symmetry. From the current data, it is unclear whether individual pulses also lack azimuthal symmetry about the beam-transmission axis, and a more detailed characterization of the X-ray focus at the CXI instrument would be valuable for a better understanding of the observed intensity distributions. We compared the observed intensities and phase variations with the pulse energies measured for individual pulses upstream of the KBs and did not find any correlation. In future experiments, it would be particularly interesting to study pulse-to-pulse fluctuations of the intensity profile and the wavefront in the focus, which were not accessible in this experiment. A method that could deliver the missing information about the characteristics and extent of the multimodal structure of the focus is multi-state ptychography (Thibault & Menzel, 2013 ▸).

### 3.6. Background characterization   

After alignment of the silicon apertures and optimization of all other focusing components, the stray light scattering (reflections from mirror, slits and apertures) was reduced to a streak in the lower left corner and flares around the beam stop. For characterization purposes, we performed a statistical analysis on 15 127 individual X-ray background (no injection) frames and calculated the mean and the variance.

**Figure 9:**
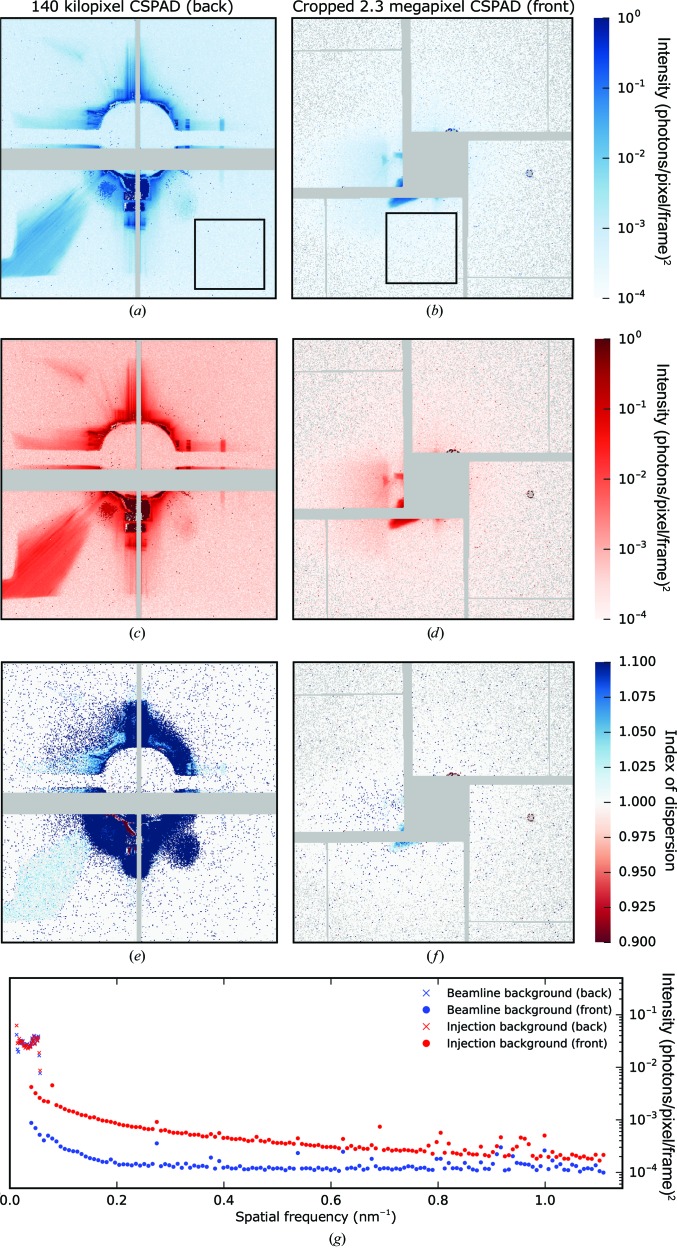
Per-pixel variance (*a*, *b*) and mean (*c*, *d*) of photon counts on the back and the centre part of the front detector after aperture alignment based on 15 127 beamline background (no injection) frames. The median photon count inside the black boxes were 4.63 × 10^−4^ (back) and 1.98 × 10^−4^ (front). The per-pixel index of dispersion (ratio of variance over mean) for the back (*e*) and the front (*f*) marks areas that follow Poisson statistics (ratio of 1) in white. Masked areas (no photons detected or bad pixels) are coloured in grey for (*a*–*f*). (*g*) Radial averages of the mean photon counts from the beamline background (*c*, *d*) in comparison to mean photon counts from injection background. Intensity values from the back detector are rescaled according to the given detector distances (relative to interaction point).

The resulting average photon counts on both detectors are shown in Figs. 9 ▸(*c*) and 9 ▸(*d*). Clean areas of the detectors had median photon counts of 4.63 × 10^−4^ and 1.98 × 10^−4^ per pixel for the back and front detectors, respectively.

In addition to the beamline background with no injection, we looked at the mean and variance of the injection background (while injecting pure buffer) based on 10 464 individual frames. A comparison of radial averages, as shown in Fig. 9 ▸(*g*), revealed a difference in signal levels for larger diffraction angles, whereas for small diffraction angles the background levels were very similar. The signal on the back detector was rescaled based on the relative distances from the interaction point. Even after rescaling there was a noticeable discontinuity, suggesting that a portion of the photons did not originate from the interaction region. This emphasizes the utility of apertures close to the interaction region to reduce background at high angles from upstream components.

By calculating the per-pixel index of dispersion (variance over mean), we managed to map out a majority of individual pixels which follow Poisson statistics (index of dispersion equal or close to 1) as shown in Figs. 9 ▸(*e*) and 9 ▸(*f*). A Poisson-like signal indicates that the background mostly fluctuated with respect to global pulse intensity variations, which is helpful when trying to build up accurate background models for use in advanced orientation-recovery and phasing algorithms.

### 3.7. Signal *versus* background   

**Figure 10:**
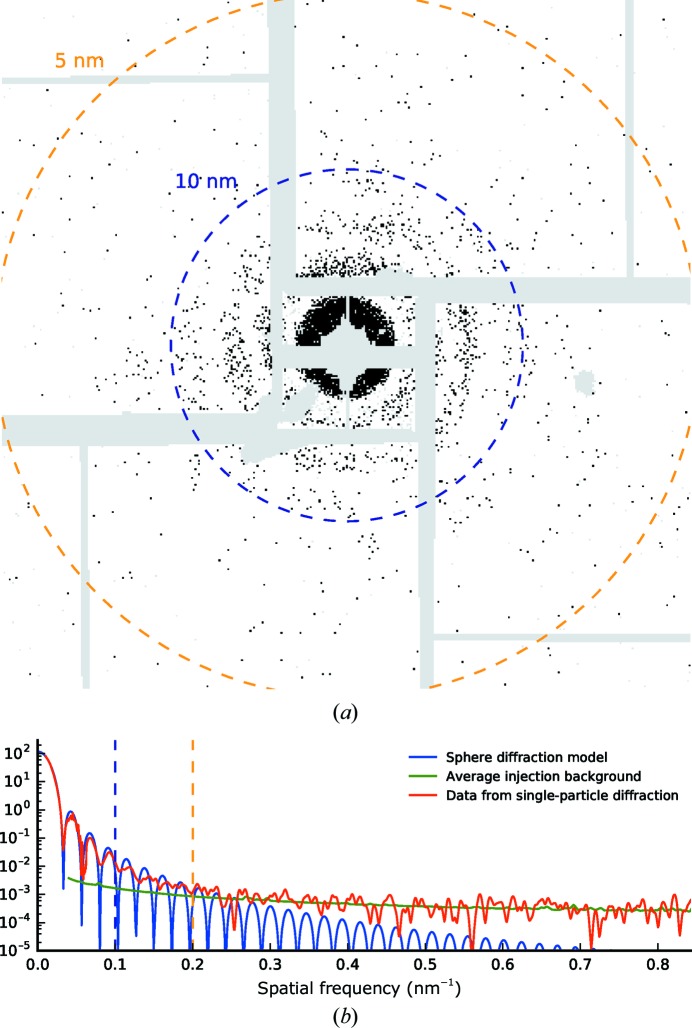
Comparison of signal from a strong single-shot diffraction pattern and average injection background. (*a*) Assembled diffraction pattern from a single shot with an estimated particle size of 43.2 nm and an incident photon intensity of 8.65 × 10^11^ photons µm^−2^. Single photons are coloured in black (the central speckle consists of multiple photons per pixel). Masked areas (no photons detected, bad pixels, strong X-ray background) are coloured grey. The image is cropped to a special frequency of 0.2 nm^−1^ at the edge. (*b*) Radial averages of the single-shot diffraction (red), the average injection background (green) and the sphere diffraction model (blue). A Gaussian filter with a kernel sigma of one pixel was applied to both traces from experimental data (red and green). Dashed rings in (*a*) and lines in (*b*) indicate full-period resolution of 5 and 10 nm.

The assembled and cropped diffraction pattern from a single strong shot is shown in Fig. 10 ▸(*a*) for a particle with an estimated size of 43.2 nm, which is close to the expected size of the sample. The estimated incident photon intensity was 8.65 × 10^11^ photons µm^−2^ and is indicative of the achievable signal level at the given experimental parameters. The sample diffraction signal matches the sphere model at lower diffraction angles and approaches the signal level of the injection background at diffraction angles above ∼0.22 nm^−1^ (Fig. 10 ▸ *b*).

The source of this background was possibly scattering from the injection gas (He) used to produce and focus the aerosol. By introducing in-chamber, post-sample apertures downstream from the interaction region, this could be mitigated, as was performed by Munke *et al.* (2016 ▸). An improved injector design could reduce the gas load in the interaction region. An alternative approach could be to extend existing algorithms for the three-dimensional assembly of single-shot diffraction patterns to reject extraneous noise by simultaneously reconstructing an *a priori* unknown background as part of the optimization problem (Loh, 2014 ▸).

### 3.8. Image reconstruction   

**Figure 11:**
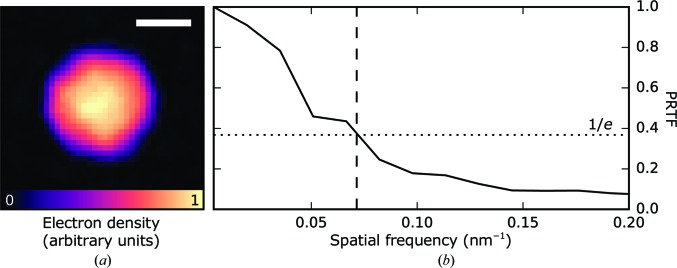
Image reconstruction based on an individual diffraction pattern (the same as shown in Fig. 10 ▸) of a sample-sized object. (*a*) Average magnitude based on 5000 independent reconstructions. The scale bar indicates 20 nm. (*b*) The phase-retrieval transfer function (PRTF) drops below 1/*e* (dotted line) at a resolution of 13.5 nm (dashed line).

For reconstructing a real-space image based on the single sample-sized diffraction pattern from Fig. 10 ▸, we recovered the phases using 5000 iterations of the HIO algorithm with β = 0.9 and 1000 iterations of the ER algorithm. Prior to phasing, the diffraction pattern was downsampled by a factor of four in each dimension. Throughout the reconstruction, we used a circular static support with a radius of 24 nm (corresponding to 12 pixels). The oversampling ratio (Miao *et al.*, 1998 ▸) of the downsampled diffraction pattern was 36. In Fig. 11 ▸, we show the magnitude of the average image reconstruction and the corresponding PRTF based on 5000 independent reconstructions. We found no evidence of missing modes (Thibault *et al.*, 2006 ▸) as the missing centre was reproducibly recovered. The PRTF dropped below 1/*e* at a full-period resolution of 13.5 nm.

## 4. Conclusion   

In this study, we attempted to transfer the success with flash X-ray imaging (FXI) using soft X-rays in a micrometre-sized focus to FXI with hard X-rays in a submicrometre focus, testing possibilities of imaging single particles of small biological samples. We aerosolized and injected our test sample: particles of the ∼40 nm *Omono River virus*. We collected a data set which enabled us to characterize the complete experimental setup and allowed us to identify targets for future development of the beamline and of the technique.

The sample-delivery system gave an overall average hit ratio of 0.83%. However, on top of a few sample-sized particles, a wide distribution of mostly spherical cluster objects (see Fig. 5 ▸) was detected. This was likely to be caused by the atomizer generating a population of larger droplets in combination with low amounts of non­volatile minor species being present. Smaller droplets could reduce the amount of trapped contaminants. For example, electrospray ionization can produce droplets which are an order of magnitude smaller in size compared with the GDVN injection system used for this experiment. This would decrease the droplet volume by a factor of 1000 and thereby reduce the impact of a salt crust or debris. An alternative atomization method could be a compound liquid jet with an outer sheath liquid of volatile buffer to aid droplet formation without contributing excess nonvolatile species (Trebbin *et al.*, 2014 ▸). In addition, online monitoring of sample injection with laser illumination could greatly aid in tuning the injection parameters (Awel *et al.*, 2016 ▸).

From a single diffraction pattern of a sample-sized particle, we succeeded in phasing and achieved a two-dimensional reconstruction of the particle to a full-period resolution of 13.5 nm. Although not suitable for a more advanced imaging analysis, the large number of spherical particles allowed us to characterize many of the experimental parameters (average beam profile, map of local phase tilts in the wavefront, level of signal and background at different diffraction angles) and provided an excellent data set for developing new algorithms for FXI. In line with recent efforts of the FXI community to make software available to others (Maia *et al.*, 2016 ▸), we shared our software tools through a public repository (https://github.com/FXIhub/cxic9714-analysis) and deposited the data in the CXIDB (https://cxidb.org).

## Figures and Tables

**Table 1:** Validation results for classification after statistical analysis of error distributions corresponding to values shown in green in Fig. 2 ▸ for diffraction centre position, particle size and intensity

Parameter	Error metric	Unit	Minimum	Maximum	Standard deviation
Horizontal centre position	*x*_fit_ − *x*_truth_	pixel	−1.46	1.05	0.10
Vertical centre position	*y*_fit_ − *y*_truth_	pixel	−0.59	1.99	0.08
Particle diameter	*d*_fit_ − *d*_truth_	nm	−0.91	3.19	0.15
Photon intensity	(*I* ^0^_fit_ − *I* ^0^_truth_)/*I* ^0^_truth_	—	−0.01	0.41	0.05

**Table 2:** Estimates for the FWHM of the reconstructed beam in the focus for different assumptions of beamline transmission The corresponding low-intensity limit (the largest distance from the beam axis) is given for each reconstruction.

Transmission (%)	FWHM (nm)	Sampled radius (µm)
1	117	0.72
10	369	2.27
20	522	3.2
30	639	3.9
