# Experimental strategies for imaging bioparticles with femtosecond hard X-ray pulses. Corrigendum

**DOI:** 10.1107/S2052252519004317

**Published:** 2019-04-09

**Authors:** Benedikt J. Daurer, Kenta Okamoto, Johan Bielecki, Filipe R. N. C. Maia, Kerstin Mühlig, M. Marvin Seibert, Max F. Hantke, Carl Nettelblad, W. Henry Benner, Martin Svenda, Nicuşor Tîmneanu, Tomas Ekeberg, N. Duane Loh, Alberto Pietrini, Alessandro Zani, Asawari D. Rath, Daniel Westphal, Richard A. Kirian, Salah Awel, Max O. Wiedorn, Gijs van der Schot, Gunilla H. Carlsson, Dirk Hasse, Jonas A. Sellberg, Anton Barty, Jakob Andreasson, Sébastien Boutet, Garth Williams, Jason Koglin, Inger Andersson, Janos Hajdu, Daniel S. D. Larsson

**Affiliations:** a Laboratory of Molecular Biophysics, Department of Cell and Molecular Biology, Uppsala University, Husargatan 3 (Box 596), SE-751 24 Uppsala, Sweden; b NERSC, Lawrence Berkeley National Laboratory, Berkeley, California, USA; c Division of Scientific Computing, Department of Information Technology, Science for Life Laboratory, Uppsala University, Lägerhyddsvägen 2 (Box 337), SE-751 05 Uppsala, Sweden; d Physical and Life Sciences Directorate, Lawrence Livermore National Laboratory, 7000 East Avenue, Livermore, CA 94550, USA; e Molecular and Condensed Matter Physics, Department of Physics and Astronomy, Uppsala University, Lägerhyddsvägen 1 (Box 516), SE-751 20 Uppsala, Sweden; f Center for Free-Electron Laser Science, DESY, Notkestrasse 85, 22607 Hamburg, Germany; g Centre for BioImaging Sciences, National University of Singapore, Singapore; h Bhabha Atomic Research Center, Mumbai 400 085, India; i Department of Physics, Arizona State University, Tempe, AZ 85287, USA; j The Hamburg Center for Ultrafast Imaging, University of Hamburg, Luruper Chaussee 149, 22761 Hamburg, Germany; k Biomedical and X-ray Physics, Department of Applied Physics, AlbaNova University Center, KTH Royal Institute of Technology, SE-106 91 Stockholm, Sweden; l ELI Beamlines, Institute of Physics, Czech Academy of Science, Na Slovance 2, 182 21 Prague, Czech Republic; m Linac Coherent Light Source, SLAC National Accelerator Laboratory, 2575 Sand Hill Road, Menlo Park, CA 94025, USA; n Brookhaven National Laboratory, 743 Brookhaven Avenue, Upton, NY 11973, USA; o Institute of Physics AS CR, v.v.i., Na Slovance 2, 182 21 Prague 8, Czech Republic

**Keywords:** X-ray diffraction, free-electron laser, flash X-ray imaging, diffraction before destruction, virus, *Omono River virus*, OmRV

## Abstract

Corrections to the article by Daurer *et al.* [*IUCrJ* (2017). **4**, 251–262] are given.

## Abstract 2

Corrigendum to the article by Daurer *et al.* [*IUCrJ* (2017). **4**, 251–262.].

The following corrections should be made in the article by Daurer *et al.* (2017 ▸).

(i) The denominator ‘3’ should not be included in equation (1); the corrected equation is:
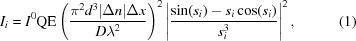



(ii) The factor of ‘2’ in equation (2) should not be there; the corrected equation is: 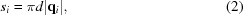



(iii) The maximum photon intensity in the last row, column 5 of Table 1 should be ‘0.25’ and not ‘0.41’.

(iv) The standard deviation of the photon intensity in the last row, column 6 of Table 1 should be ‘0.03’ and not ‘0.05’.
